# Setting, Strength, and Autogenous Shrinkage of Alkali-Activated Fly Ash and Slag Pastes: Effect of Slag Content

**DOI:** 10.3390/ma11112121

**Published:** 2018-10-29

**Authors:** Marija Nedeljković, Zhenming Li, Guang Ye

**Affiliations:** Microlab, Faculty of Civil Engineering and Geosciences, Delft University of Technology, Stevinweg 1, 2628 CN Delft, The Netherlands; Z.Li-2@tudelft.nl (Z.L.); G.Ye@tudelft.nl (G.Y.)

**Keywords:** alkali-activated pastes, slag content, workability, setting, compressive and flexural strength, autogenous shrinkage

## Abstract

The engineering properties of alkali activated materials (AAMs) mainly depend on the constituent materials and their mixture proportions. Despite many studies on the characterization of AAMs, guidelines for mixture design of AAMs and their applications in engineering practice are not available. Extensive experimental studies are still necessary for the investigation of the role of different constituents on the properties of AAMs. This paper focuses on the development of alkali-activated fly ash (FA) and ground granulated blast furnace slag (GBFS) paste mixtures in order to determine their suitability for making concretes. In particular, the influence of the GBFS/FA ratio and liquid-to-binder (l/b) ratio on the slump, setting, strength, and autogenous shrinkage of the alkali activated pastes is studied.It is shown that fresh properties largely depend on the type of precursor (GBFS or FA). The slump and setting time of GBFS-rich pastes was significantly reduced. These pastes also have higher compressive strength than FA-rich pastes. The study identifies important practical challenges for application of the studied mixtures, such as the behavior of their flexural strength and high amplitudes of autogenous shrinkage of GBFS-rich mixtures. Finally, the optimum GBFS/FA ratio for their future use in concretes is recommended.

## 1. Introduction

Cement-based concrete is used as a main material for infrastructures and buildings. Concrete structures have been shown to be safe and durable, and this was one of the most important criteria for their design and use in the past. Nowadays, however, the sustainability requirements present the main challenge for the concrete construction industry [1,2]. More specifically, there are several aspects that should be considered and improved in the current cement-based concrete production. Most important are high CO_2_ emissions and high energy consumption due to CaCO_3_ calcination for cement production [3,4,5]. Currently, the cement industry accounts for approximately 5–8.6% of the global CO_2_ emissions [1,6,7]. The regulations on the permissible level of emission of CO_2_ make it necessary to innovate and improve the energy efficiency and provide environmentally friendly alternatives for the cement industry. To fulfil sustainability criteria of reduced CO_2_ emissions, low CO_2_-emission cements are needed. Furthermore, production of waste from various industrial processes is a concern as most of the waste cannot be landfilled and thus presents a burden for both its producer and the environment [8]. Replacement of ordinary Portland cement (OPC) with supplementary cementitious materials (SCMs) [9] is one of the possibilities to reduce the environmental impact of OPC production.

Commonly, SCMs are by-products from industrial processes, such as fly ash (FA) from coal-fired electricity production and ground granulated blast-furnace slag (GBFS) from steel production [10]. The extent of replacement of OPC is limited by the composition and reactivity of the SCMs [9]. In fact, FA and GBFS can completely replace OPC by taking advantage of alkali activation [4]. In this case, alkali activated materials (AAMs) are produced [11].

Today a lot of research has been devoted to the development and characterization of AAMs [12,13,14,15,16,17,18,19,20,21,22,23,24,25,26,27,28,29,30,31,32,33,34,35] to promote their use because of their “green” nature. So far, AAMs are increasingly used for non-structural applications. The widespread use of AAMs in structural applications is limited by lack of standards and understanding of their long-term behaviour [11,36,37]. Furthermore, a number of open questions still exist with regard to mixture design of AAMs. 

In the mixture design of AAMs, the most-used raw materials are FA, GBFS, and metakaolin. Factors that are broadly studied for mixture design of alkali-activated systems are the properties of:

- raw materials (chemical composition [38], reactivity, surface area [39], particle size distribution [40], and loss on ignition) and
- alkaline activators (alkali concentration, viscosity of activator, pH of activator, water-to-solid ratio, modulus, and dosage of alkaline activator).

A relatively high proportion of silica (SiO_2_), or the sum of SiO_2_, alumina (Al_2_O_3_), and iron (III) oxide (Fe_2_O_3_), is needed to ensure that sufficient potentially reactive glassy material is present in FA. When FA with a high CaO content is activated in an alkaline environment, the effect of a high calcium concentration typically leads to the acceleration of the rate of reaction. In a pozzolanic reaction between FA and Ca(OH)_2_ or calcium silicate phases in OPC-based paste, the early reaction may be so rapid that it will be unsuitable for applications that require longer workability or setting time. Therefore, FA Class F is preferred in OPC-based materials and alkali activated binders due to the high content of amorphous aluminosilicate phases and low CaO content [41].

Fernandez-Jimenez et al. [40] observed that the fineness of FA has an important effect on the development of the compressive strength of alkali activated systems. It was reported that when the mean particle size was lower than 45 µm, strength increased remarkably, reaching 70 MPa after 1 day. Beside the mean particle size, Van Jaarsveld et al. [15] reported that the surface charge on the FA particle affects the initial setting properties of an alkali activated FA. 

Challenges regarding casting, such as fast setting time and harsh workability, are well-known for alkali-activated GBFS-rich pastes [42]. This behaviour originates from the amorphous nature of GBFS and high Na_2_O concentration of alkaline activator normally used for the activation of GBFS [43,44,45]. The development of the compressive strength of pure GBFS-based pastes also depends significantly on the type of activator [46]. For instance, GBFS pastes activated with NaOH develop a higher compressive strength at early ages compared to GBFS pastes activated with hydrous sodium metasilicate Na_2_SiO_3_·5H_2_O. In contrast, these pastes have higher compressive strength than ones with NaOH at later ages [46].

The importance of flexural strength has been demonstrated by Ma [33] and Wardhono et al. [47]. It was observed that the flexural strength of AAMs varied widely with the type of precursor (FA or GBFS) and composition of the alkaline activator. It was reported that at early ages, the flexural strength increased; however, it decreased for some of the investigated mixtures at later ages [33]. More recently, Wardhono et al. [47] reported an increase of the flexural strength for alkali-activated FA-based concrete over time, while the reduction of flexural strength was observed for alkali-activated GBFS-based concrete. In both studies, References [33,47], compressive strength increased irrespective the mixture type.

Although there has been extensive research regarding the factors influencing the workability, setting time, and development of the mechanical properties of pure alkali-activated FA and pure alkali-activated GBFS, little attention has been paid to these properties of blended FA and GBFS mixtures. Furthermore, regarding the volume change, the autogenous shrinkage may be more problematic than drying shrinkage for alkali activated mixtures [48,49]. The autogenous shrinkage develops fast at an early age when the strain capacity of the concrete remains low and may lead to microcracking. In order to better understand material properties of alkali activated binders, the present paper focuses on the influence of the GBFS/FA ratio and liquid-to-binder (l/b) ratio on the workability, setting time, compressive strength, flexural strength, and autogenous shrinkage of the alkali activated pastes. A set of different mixtures for selecting an optimum GBFS/FA ratio for concrete design was studied. In addition, an attempt was focussed on identifying the cause of wide deviations for flexural strength results. 

## 2. Materials and Paste Mixture Design

### 2.1. Raw Materials

The raw materials used in this study were GBFS from ORCEM (Moerdijk, The Netherlands) and FA from VLIEGASUNIE BV (Culemborg, The Netherlands). GBFS had a specific gravity of 2890 kg/m^3^, while FA had a specific gravity of 2440 kg/m^3^. The chemical composition of the GBFS and FA determined with X-ray fluorescence (XRF) is given in Table 1. XRF measurements were carried out with a PANalytical’s Epsilon 3^XLE^ spectrometer (Malvern Panalytical Ltd., Malvern, UK) equipped with a rhodium X-ray source, the silicon-drift detector with a 135 eV resolution at 5.9 keV/1000 cps. Two to three grams of FA/GBFS powder was poured in a 32 mm spectra cup fitted with a bottom of stretched 4 µm prolene film held with a concentric ring. Sulphur (S) was determined with an Eltra Sulphur analyser (ELTRA GmbH, Haan, Germany). Loss on ignition (LOI) was determined with a LECO Thermogravimetric Analyser (TGA701) (LECO Corporation, St. Joseph, MI, USA). The negative LOI value in GBFS is related to the oxidation of sulfur rich compounds in the GBFS. The LOI of FA is related to the unburned carbon content.

The GBFS had a higher proportion of CaO and MgO and lower proportions of SiO_2_, Al_2_O_3_, and Fe_2_O_3_ compared to FA. The basicity coefficient (K_b_ = (CaO + MgO)/(SiO_2_ + Al_2_O_3_)) for GBFS was 0.98, which nearly complies with the neutral type of GBFS (K_b_ = 1.0). The neutral GBFS is preferred for alkali activation as shown by Chang [43]. The hydration modulus according to a formula proposed in Reference [43] (HM = (CaO + MgO + Al_2_O_3_)/SiO_2_) of GBFS was 1.73. This was higher than the required value of 1.4 for good hydration properties of GBFS [43]. The FA complied with Class F FA (EN 450, ASTM C618) since it had low CaO content (≤10% reactive CaO, EN 450) and SiO_2_ + Al_2_O_3_ + Fe_2_O_3_ ≥ 70%.

**Figure 1:**
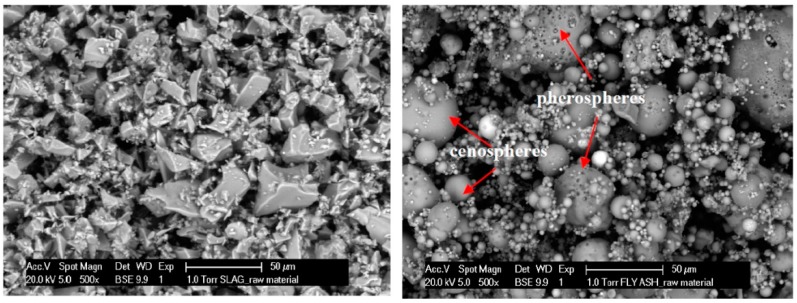
ESEM-BSE images of GBFS (**left**) and FA (**right**) particles.

The particle shape of GBFS and FA was studied with a Philips-XL30-ESEM environmental scanning electron microscope (FEI, Eindhoven, the Netherlands) in backscattered electron mode (ESEM-BSE). The raw GBFS particles had clear edges and angles as shown in Figure 1. This was due to inter-impacting and inter-rubbing between steel balls in the ball mill as GBFS needs to be processed with a ball mill. On the other hand, the raw FA particles consisted of individual and agglomerated spheres of different sizes. A large quantity of FA spheres were hollow, known as cenospheres or floaters, which were very light and tended to float on water surface [10]. FA also contained small spherical particles within a large sphere, called pherospheres [10] as indicated by red arrows in Figure 1. The external surfaces of the solid and hollow spherical particles of low-CaO FA, as FA in this study, were generally smoother than those of high-CaO FA, which may have surface coatings of material rich in CaO [10].

**Figure 2:**
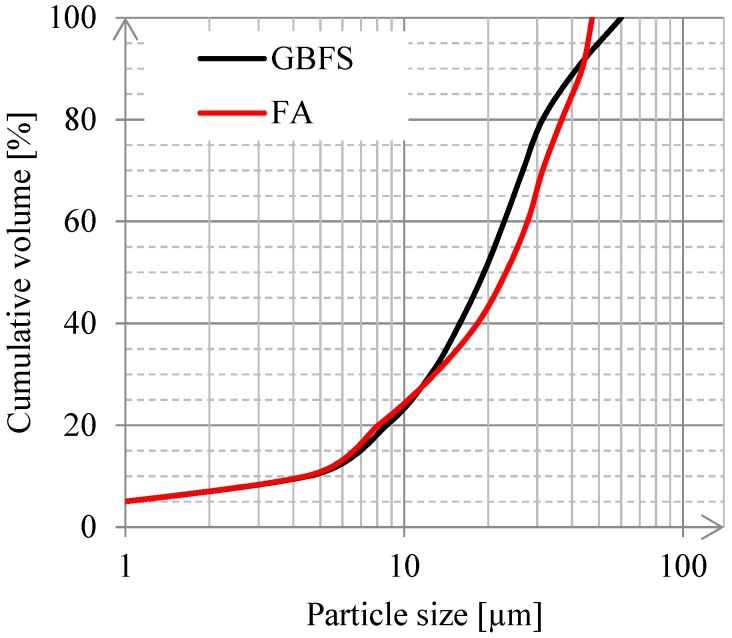
Particle size distributions of GBFS and FA as measured with a laser diffraction analyser.

Figure 2 shows the particle size distributions of GBFS and FA, which were measured with an EyeTech Laser diffraction analyser (Ankersmid International, Oosterhout, The Netherlands). An external ultrasonic bath was used for the deagglomeration of the particles, in order to increase the dispersion efficiency. The d_50_, which represents the particle size of a cluster of particles, was 19 μm for GBFS, while for FA, d_50_ was 21 μm. According to the literature, the dissolution of GBFS is dominated by small particles. Particles >20 µm react slowly, while particles <2 µm react completely after 24 h in blended cements and in alkali-activated binders [50,51].

**Figure 3:**
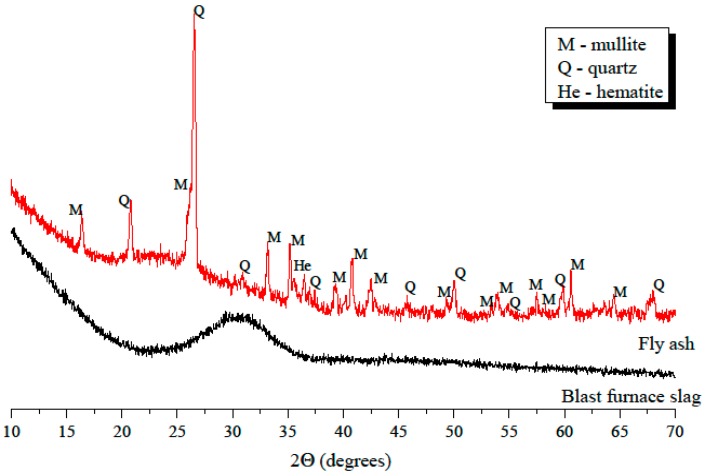
XRD diffractograms for GBFS and FA.

The mineralogical composition of GBFS and FA was studied with X-ray diffraction (XRD). XRD diffractograms of GBFS and FA were acquired using a Philips PW 1830 powder X-ray diffractometer (FEI, Eindhoven, The Netherlands), with Cu Kα (1.789 Å) radiation, tube setting 40 kV and 40 mA, step size of 0.030°, rate of 2.0 s per step, and 2θ range of 10–70°. The amorphous phase was dominant in GBFS (see Figure 3). The presence of the amorphous phase can be recognized by the broad hump centred around 31° 2θ. In contrast to GBFS, FA also contained crystalline phases, such as mullite, quartz, and hematite, alongside the amorphous phase.

### 2.2. Alkaline Activator

The alkaline activator composition was selected and modified based on the work of Marinković et al. [52]. The Na_2_O concentration of alkaline activator was reduced from 9.5 wt% to 4.8 wt%, and modulus (n = SiO_2_/Na_2_O) was reduced from 1.91 to 1.45. In contrast to the previous studies [52,53], the Na_2_O concentration was reduced significantly for this study because of environmental reasons and high costs for the alkaline compounds. Furthermore, the composition of the alkaline activator was set according to recommendations from earlier studies [39,54,55]. Wang et al. [39] demonstrated that the sodium silicate solutions with moduli of 1–1.5 gave the best strengths regardless of curing conditions and type or fineness of GBFS. The authors also suggested that the optimum Na_2_O concentration was likely to be within the range of 3.0–5.5% Na_2_O by the GBFS weight [39,55]. Finally, the alkaline activator was synthetized by mixing anhydrous pellets of sodium hydroxide with deionized water and sodium silicate solution in a 1:1 weight proportion. A 4 M sodium hydroxide solution was used. The chemical composition of sodium silicate solution was: 27.5 wt% SiO_2_, 8.25 wt% Na_2_O, and 64.25 wt% H_2_O.

### 2.3. Mixture Design

In this study, the baseline was a constant liquid-to-binder ratio, while the FA/GBFS ratio was varied in the mixture design of the pastes. Note that mixtures might not have had an optimized activator composition for a specific FA/GBFS ratio. An additional requirement was that all mixtures were suitable for casting. The mixtures included individual and blended systems. The individual systems were alkali-activated FA (S0) and alkali-activated GBFS (S100). Blended systems were alkali-activated FA/GBFS mixtures with the following ratios: 70:30, 50:50, and 30:70 wt%, named S30, S50, and S70, respectively. In addition to different FA/GBFS proportions, the liquid/binder (where “binder” is defined as the FA and/or GBFS content) mass ratio was varied, as 0.4 and 0.5, to examine the effect of different liquid-to-binder ratios on the workability and setting time. All mixtures were designed with the alkaline activator composition defined in Section 2.2. Alkaline activator was prepared 24 hours prior to casting in order to cool down to ambient temperature. The synthesis of alkaline activator initially releases heat and 24 h is needed for heat dissipation and dissolution of NaOH in the solution. The pH of the activator was 14, as measured using an 827 Metrohm pH meter (Metrohm, Herisau, Switzerland). The detailed mixture design is given in Table 2. The workability and setting time were compared between alkali-activated pastes and the ordinary Portland cement (CEM I 42.5 N) paste.

### 2.4. Experimental Programme

#### 2.4.1. Workability

The properties of fresh mixtures were determined by testing their workability and the setting time. The workability of mixtures was tested using the mini-slump spread test as recommended by Tan et al. [56]. The raw material was first premixed for 3 min prior to mixing with the alkaline activator. For each mini-slump test, 400 g of raw material was mixed with the alkaline activator in a glass cylindrical container (diameter 10 cm and height 15 cm) for 2 min by hand. The fresh mixture was poured into a slump cone with a top inner diameter of 36 mm, a bottom inner diameter of 90 mm, and a height of 90 mm. The cone was placed in the center of a square smooth glass plate. After filling it with the fresh mixture, the cone was lifted and the mixture subsided. The average spread of the mixture, as measured along the two diagonals and two median directions, was recorded. 

#### 2.4.2. Setting Time

With the addition of the alkaline activator to the raw materials (FA/GBFS) the chemical reaction started and the alkali-activated paste began to stiffen, accompanied by heat release. In cement-based pastes, the setting was a percolation process. In this process, isolated or weakly-bound particles are connected through the formation of reaction products so that solid paths are formed in the hardening pastes [57]. Hence, the setting of pastes will depend on factors that affect the connectivity between particles such as the liquid-to-binder ratio [58]. It is assumed that the same mechanism of setting was valid for alkali-activated pastes. However, different setting times were expected for alkali-activated pastes. The initial and final setting times of the alkali-activated FA and GBFS pastes were measured using an automatic recording Vicat needle apparatus according to NEderlandse Norm-European Norm (NEN-EN) 196-3 [59]. In practice, the initial setting indicates the loss of workability and the beginning of the stiffening of the paste or concrete [60]. Setting time tests were conducted at 20 °C and 50% relative humidity (RH). Pastes were prepared according to the mixture design in Table 2 and cast in the same way as for the workability tests. The fresh mixtures were placed in the standard truncated cone (40.0 ± 0.2 mm deep with internal diameters at top and bottom of 70 ± 5 mm and 80 ± 5 mm, respectively). According to the NEN-EN 196-3, the initial setting time is the time elapsed between “zero time” (the time when the alkaline activator is mixed with raw material) and the time at which the distance between the needle and the base plate was 6 ± 3 mm. The final setting time was the elapsed time, measured between the “zero time” and the time when the needle first penetrated only 0.5 mm into the paste.

#### 2.4.3. Calorimetric Measurements

When FA and GBFS are in contact with the alkaline activator, FA and GBFS react and consequently generate heat. The heat evolution was investigated using isothermal conduction calorimetry in accordance with ASTM C1679 [61]. All raw materials were preconditioned at an ambient temperature of 20 °C to avoid any temperature difference with the measurement temperature (20 °C). The mixtures were prepared by mixing the raw material and alkaline activator externally. About 3 min later, the mixture is placed into the calorimeter (TAM-Air-314). Two samples were measured simultaneously per mixture. The amount of heat release was recorded and the cumulative heat was calculated up to one week.

#### 2.4.4. Mechanical Properties

To investigate the effect of different FA/GBFS and liquid-to-binder ratios on the development of the mechanical properties of pastes, flexural and compressive strength were tested. A HOBART mixer was used for mixing 3-liter batches. After premixing the raw material, the alkaline activator was added at low speed mixing. Mixing continued at low speed for 1 min and for 2 min at high speed. The fresh mixtures were cast in steel prisms molds (40 × 40 × 160 mm^3^). The molds were first covered with a thin layer of oil as a demolding agent. Subsequently, filled prisms were covered with a thin plastic sheet. Samples were demolded 24 h after casting and further cured unsealed in a fog room at 20 °C and RH ≈99%.

First, the three-point flexural bending test was performed on 40 × 40 × 160 mm^3^ specimens according to the NEN-EN-196-1 [62]. Three specimens were tested per age. Two halves of the specimen were then used for testing the compressive strength. The compressive strength was calculated as an average value of six samples. Then, the mean values and standard deviations were calculated for each set of data.

#### 2.4.5. Autogenous Shrinkage

The autogenous shrinkage was tested using the corrugated tube method. The measurements started after the final setting time of the pastes (determined using a Vicat needle test), according to the ASTM standard C1698-09. Three corrugated tubes of 440 mm in length and 28.5 mm in diameter were tested for each mixture. The specimens and test instrument were immersed in glycol in a box where the temperature was kept at 20 ± 0.1 °C with the help of a cryostat. The autogenous shrinkage of specimens was recorded every 5 min using linear variable differential transformers (LVDTs). The autogenous deformation of parallel samples had a similar trend with a deviation of less than 50 microstrains; therefore, only one typical curve for each mixture will be presented.

## 3. Results and Discussion

### 3.1. Workability

**Figure 4:**
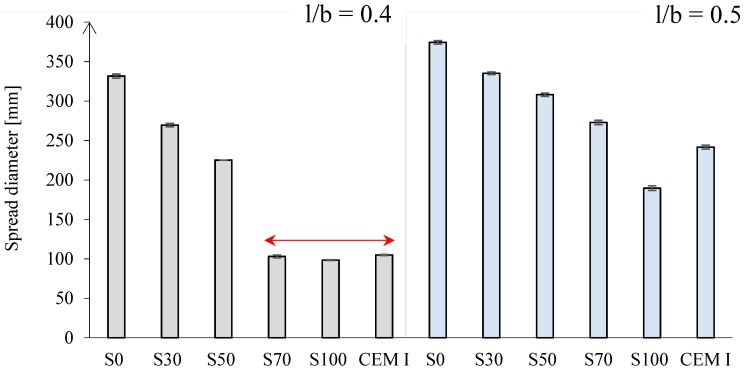
Mini-slump spread diameter of alkali-activated pastes and CEM I 42.5 N paste for l/b = 0.4 (**left**), and l/b = 0.5 (**right**).

The flow of the fresh paste mixtures was reduced with increasing GBFS content. Figure 4 shows the loss of workability for mixtures S70, S100, and CEM I with l/b = 0.4 (indicated by the red arrow). The high specific surface area and high chemical activity of GBFS required a larger amount of water than FA particles [10]. Hence, the workability and the setting time, as shown in the next section, decreased for GBFS-rich pastes. With the increase of GBFS, the liquid demand clearly increases in accordance with research of Reference [10]. Therefore, in order to maintain good consistency of the mixture, the l/b ratio was changed to 0.5. The spread diameter increased for all pastes with l/b = 0.5 compared to pastes with l/b = 0.4.

**Figure 5:**
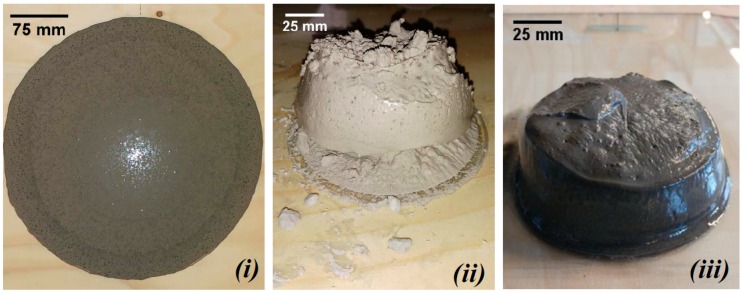
The mini-slump spread test for (***i***) S0 (alkali-activated FA paste), (***ii***) S100 (alkali-activated GBFS paste), and (***iii***) CEM I 42.5 N paste for l/b = 0.4.

**Figure 6:**
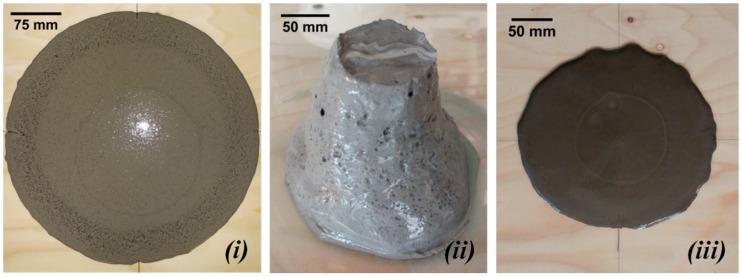
The mini-slump spread test for (***i***) S0 (alkali-activated FA paste), (***ii***) S100 (alkali-activated GBFS paste), and (***iii***) CEM I 42.5 N paste for l/b = 0.5.

**Figure 7:**
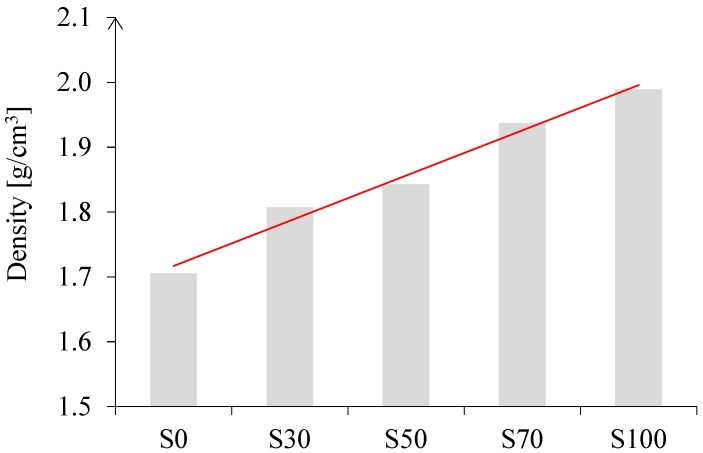
Density of the pastes S0, S30, S50, S70, and S100 with l/b = 0.5 in the fresh state.

In cement-based pastes, the water content has a dominant role in controlling workability [10], as demonstrated for CEM I pastes (see Figure 5*iii* and Figure 6*iii*). In contrast, for alkali-activated pastes, not only the liquid-to-binder ratio, but also the particle shape has a significant influence on the spread diameter. The replacement of GBFS by the same mass of FA improved the workability. The spherical shape and smooth glassy surface of FA particles (Figure 1) promote sliding of the particles. This reduced frictional forces between angular particles, which is known as the “ball bearing effect” [63]. Furthermore, the addition of FA improved the flowability due to the packing effect. The packing density of the paste increased and the water retained inside the particle flocs decreased. This was again due to the spherical shape of FA, which minimized the particle’s surface to volume ratio, resulting in a low fluid demand. A spherical shape, out of all 3-D shapes, provides the minimum surface area for a given volume [64]. In addition, a lower specific gravity of FA (2.44 g/cm^3^) than that of GBFS (2.9 g/cm^3^) or CEM I (3.0 g/cm^3^) contributed to higher flowability of the FA/GBFS pastes. This was supported by the measurements of the density of pastes in a fresh state. The fresh state density was evaluated through the weight determination of a specific paste volume. The results are shown in Figure 7. It can be seen that the density increased with increasing GBFS content. Consequently, the flowability of the GBFS-containing pastes decreased (see Figure 5*i* and Figure 6*i*).

A similar spread diameter was measured for both alkali-activated FA pastes, with l/b = 0.4 and l/b = 0.5 (Figure 4, paste S0). In contrast to alkali-activated FA paste (S0), alkali-activated GBFS paste (S100) exhibited a higher consistency (compare Figure 5*i*,*ii*). The behaviour of alkali-activated GBFS (S100) in terms of consistency is very similar to the CEM I paste (Figure 5*iii*). As for alkali-activated GBFS (S100), a higher l/b ratio contributed to a stable slump and better filling of the cone without improvement of the flowability (Figure 6*ii*). In contrast to paste S100, the workability of CEM I paste was significantly modified, whereas the higher l/b ratio enabled better flowability (Figure 6*iii* compared to Figure 5*iii*). 

### 3.2. Setting Time

**Figure 8:**
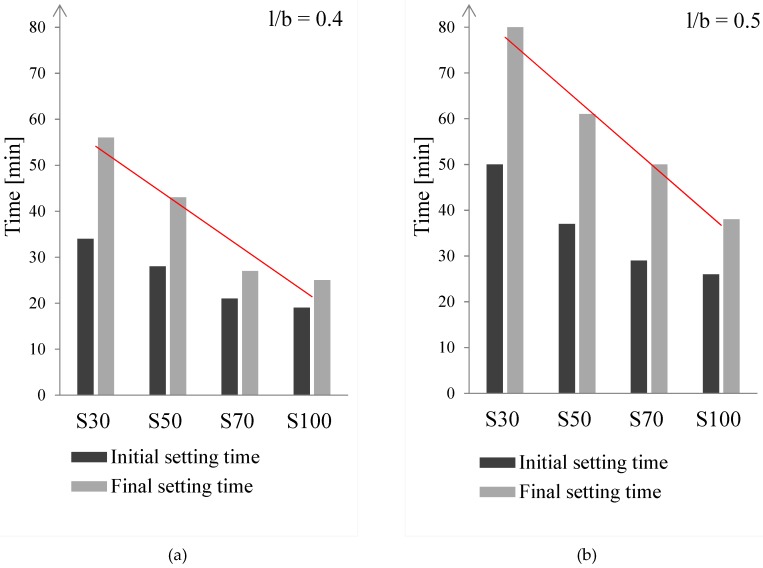
Initial and final setting time of pastes with different l/b ratios: (**a**) l/b = 0.4, and (**b**) l/b = 0.5. The setting time for S0 paste was not plotted due to very long initial setting times (for l/b = 0.4 the initial setting time was 6 h and for l/b = 0.5, it was 14 h).

The influence of the l/b ratio and GBFS content on the initial and final setting times of alkali-activated pastes are illustrated in Figure 8. In cement-based materials, setting is known as stiffening without significant development of compressive strength, which usually occurs within a few hours [65]. However, for alkali activated FA/GBFS pastes, it lasts 25–80 min (Figure 8). The setting time of alkali activated pastes is noticeably affected by the l/b ratio of the pastes and GBFS content. Both the initial and final setting time increased as the l/b ratio increased from 0.4 (Figure 8a) to 0.5 (Figure 8b). A negative linear relationship can be seen in Figure 8b between the final setting times and increase of the GBFS content. The alkali-activated FA (S0) paste had a relatively long initial setting time due to the slow rate of chemical reaction at a low ambient temperature [66]. The initial setting time for the alkali-activated FA (S0) paste with l/b = 0.4 was 6 h, while with l/b = 0.5, it was 14 h (not plotted in the Figure 8). As for CEM I 42.5 N (w/c = 0.5) paste, the initial setting is within 6 to 7 h according to the literature [67].

Both initial and final setting time decreased with an increase of GBFS content, while all the final setting times were less than 80 min. Given that the surface area of GBFS (2.54 m^2^/g) was higher than of FA (1.73 m^2^/g), and the content of the amorphous phase in GBFS (99 wt%) was higher compared to FA (68 wt%), it was expected that when dissolution took place, the GBFS would be more reactive than FA. In addition, the presence of soluble silica affected the reaction kinetics by enhancing the condensation process of dissolved GBFS. This resulted in stiff paste mixtures for S100, as demonstrated in Figure 5*ii* and Figure 6*ii*. Consequently, the apparent viscosity of the fresh pastes with more GBFS was observed to be higher than that of pastes that contain more FA, leading to a shorter setting time for GBFS-rich pastes. This effect was demonstrated in the mixture even for smaller amount of GBFS, i.e., below 30 wt% GBFS, as reported by Nath et al. [44].

To illustrate the effect of the liquid-to-binder ratio and binder composition on the setting time, final setting times for pastes S30 and S100 were compared. The final setting time was 25 and 40 min for pure alkali-activated GBFS pastes with l/b = 0.4 and l/b = 0.5, respectively, while the final setting time was 55 and 80 min for paste S30 with l/b = 0.4 and l/b = 0.5, respectively. Due to the use of highly alkaline solutions (pH > 14), the alkaline activation promoted the rapid precipitation of reaction products from the pore solution in GBFS-rich pastes. The fine particle size distribution and high specific surface area of GBFS, such as in the paste S100, accelerated the hydration process, which had a “thickening” effect on the pastes. Hence, the apparent viscosity increased and the fresh grouts became more viscous [68]. This promoted strength growth at early ages, as will be demonstrated in Section 3.4. The effect of GBFS content on the initial setting time of the pastes (l/b = 0.5) will be further examined in the next section by studying the rate of heat release. 

### 3.3. Isothermal Heat Release of Pastes

**Figure 9:**
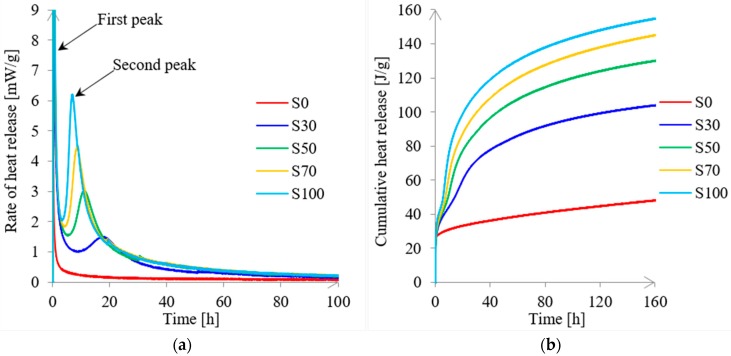
Calorimetric curves for the rate of heat release (**a**) and cumulative heat release (**b**) for alkali-activated pastes with l/b = 0.5.

The calorimetric heat release curves of alkali-activated pastes are shown in Figure 9. Figure 9a shows the rate of heat release, while Figure 9b shows the cumulative heat release for alkali-activated FA/GBFS pastes with l/b = 0.5. The calorimetric curves for the rate of heat release for all pastes have two characteristic peaks except for paste S0. Similar calorimetric curves are observed for GBFS activated by sodium silicate solution [27]. The first peak was observed within the first few minutes of reaction. It corresponded to the wetting and partial dissolution of FA and GBFS. The second broad peak occurred between 6.5 h and 17 h, which corresponded to the formation of reaction products [69]. The second peak did not appear for paste S0, meaning that none or a very little amount of reaction products were formed in this paste. 

The rate of heat release shows how fast the dissolution of FA and GBFS was (see Figure 9a). The rate varied among pastes. It was higher for GBFS-rich pastes due to higher reactivity of GBFS. Consequently, the cumulative heat release curves for alkali-activated pastes indicated more heat release with increasing GBFS content (Figure 9b). The setting times of the alkali-activated mixtures agreed well with calorimetry results. With increasing GBFS content, the setting time became shorter (Figure 8) due to the fast formation of a large amount of reaction products in the GBFS-rich pastes (Figure 9a).

### 3.4. Mechanical Properties

#### 3.4.1. Compressive Strength

S100 Paste

**Figure 10:**
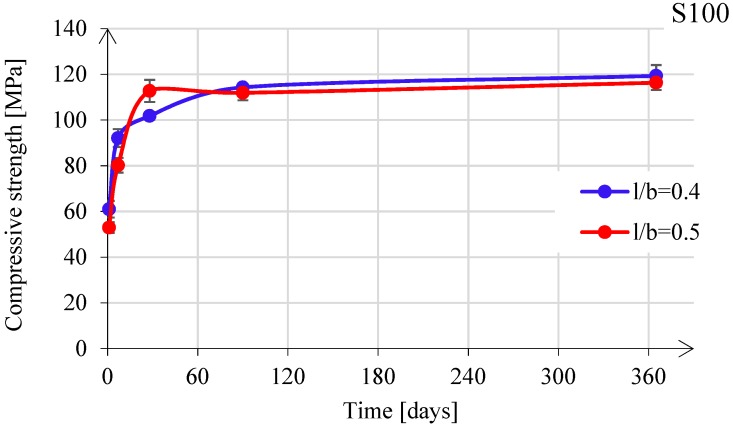
Compressive strength development for S100 paste.

Figure 10 shows the influence of the l/b ratio on compressive strength for paste S100. It can be seen that for both l/b ratios (0.4 and 0.5), compressive strength values were similar. The maximum compressive strength for paste S100 was obtained after 14 days (see Figure 10). An extended curing time in the fog room did not contribute to a further increase of the compressive strength. In contrast, the curing time for obtaining the maximum compressive strength in OPC-based paste is around 60 days (84.5 MPa), as reported by Chindaprasirt et al. [70]. The comparison was based on similar water-to-binder ratio (0.35 for OPC-based paste and 0.38 for paste S100). Table 3 presents an overview of differences regarding gel properties in two pastes: S100 and OPC-based paste. The brittle behaviour and high compressive strength of alkali-activated GBFS-rich pastes was assumed to be the result of the combined effect of low porosity and dense packing of grains. Thomas et al. [71] reported that the specific surface area of the alkali-activated GBFS pastes is about 25% higher than that of OPC-based paste cured under the same conditions. Furthermore, the molar volume calculations indicated that the atomic packing density is significantly higher in gel phases of alkali activated GBFS paste than in OPC-based paste. Therefore, the presence of nanoparticulate building units in brittle materials gives superior mechanical strength with regard to the bulk material, as shown by Knudsen [72].

FA/GBFS Blended Pastes

**Figure 11:**
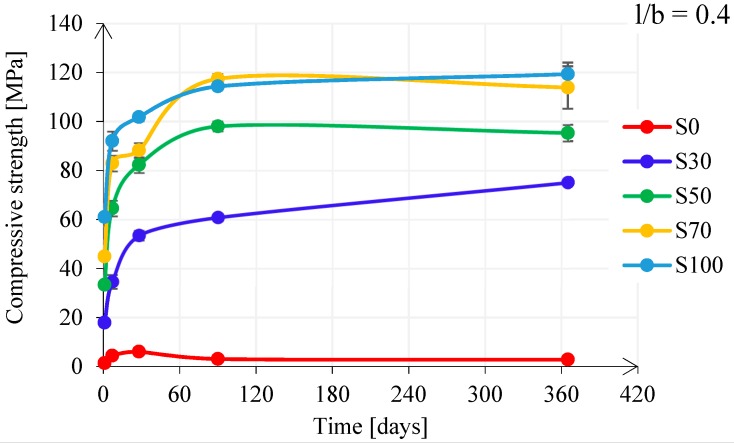
Compressive strength development for pastes with l/b = 0.4.

Figure 11 shows the influence of the FA/GBFS ratio on the compressive strength development for pastes with l/b = 0.4. The results of the compressive strength imply that the addition of a small amount of calcium into FA systems significantly improved their mechanical properties. By increasing the GBFS content, the compressive strength increased during the first 90 days. After 1 year, the compressive strength was nearly the same for GBFS-rich pastes: S70 and S100. A slight decrease of compressive strength for pastes S50 and S70 could be observed at ages > 90 days. However, the level of decrease of the compressive strength was within the bandwidth of the standard deviation. It can also be seen from Figure 11 that the strength development at 1 year had stagnated for GBFS > 50 wt%. This suggests that dissolution of raw materials between 90 days and 365 days was hindered. This is further confirmed in Reference [73] by comparing the Na^+^ concentration from the pore solution between 28 and 365 days. The Na^+^ concentration was not changed for pastes S70 and S100, supporting the strength stagnation. The results for compressive strength of alkali activated pastes were in accordance with calorimetric response of pastes. Alkali-activated FA-rich pastes have reduced cumulative heat release due to the slower reaction of FA in the pastes compared to GBFS in GBFS-rich pastes. Consequently, FA-rich pastes had a lower strength development.

S0 paste

For paste S0 (Figure 11), strength development was significantly limited by the low Na_2_O concentration of the alkaline activator and due to the applied curing conditions, which for all pastes were the same (unsealed curing conditions, 99% RH, 20 °C). Normally, FA requires much higher Na_2_O concentration and elevated curing temperatures (>40 °C) in order to be dissolved [33]. This is consistent with very low heat release of paste S0, shown in Figure 9.

**Figure 12:**
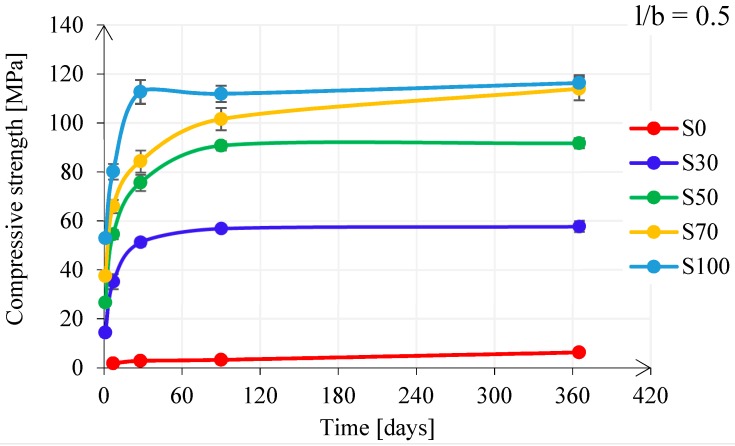
Compressive strength development for pastes with l/b = 0.5.

Figure 12 presents the compressive strength development for pastes with l/b = 0.5. The compressive strength was slightly lower than for the pastes with l/b = 0.4, suggesting that the increase of the l/b ratio did not significantly affect compressive strength development. The tendency of the strength development followed the tendency of the cumulative heat release curves for the first 7 days (Figure 9). Higher cumulative heat release of the GBFS pastes indicated their high compressive strength compared to FA-rich pastes. In Figure 12, it can also be seen that with 70 wt% GBFS in the mixture, the compressive strength after 1 year reached almost the same compressive strength as with 100 wt% GBFS. Compared to respective pastes with l/b = 0.4, this time was 90 days. This implies that pastes with l/b = 0.4 had a stagnation of the compressive strength, in particular for paste S100, as indicated in Figure 10 and Figure 11. The compressive strength development for pastes with l/b = 0.5 (see Figure 12) showed that the studied pastes had satisfactory strength. 

#### 3.4.2. Flexural Strength

**Figure 13:**
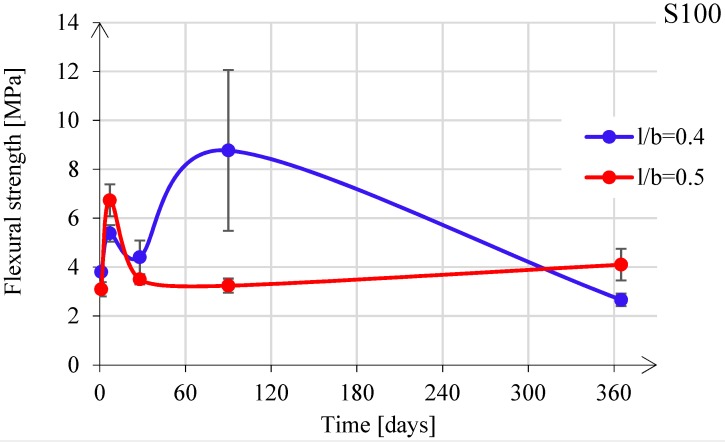
Flexural strength development for S100 paste.

Figure 13 shows the influence of the l/b ratio on the flexural strength development for paste S100. The flexural strength increased during the first 7 days, whereas it decreased to a minimum at 28 days, for both l/b = 0.4 and l/b = 0.5. At a later age, i.e., at 90 days, the flexural strength for l/b = 0.4 increased, while for l/b = 0.5, it had no further change. Between 90 and 365 days, it decreased again for l/b = 0.4, while it increased for l/b = 0.5, resulting in a strength of 3.5 and 4.0 MPa, respectively.

**Figure 14:**
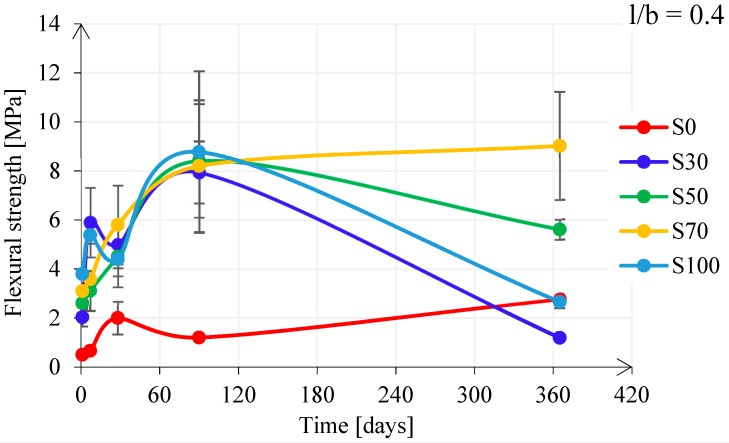
Flexural strength development for pastes with l/b = 0.4.

**Figure 15:**
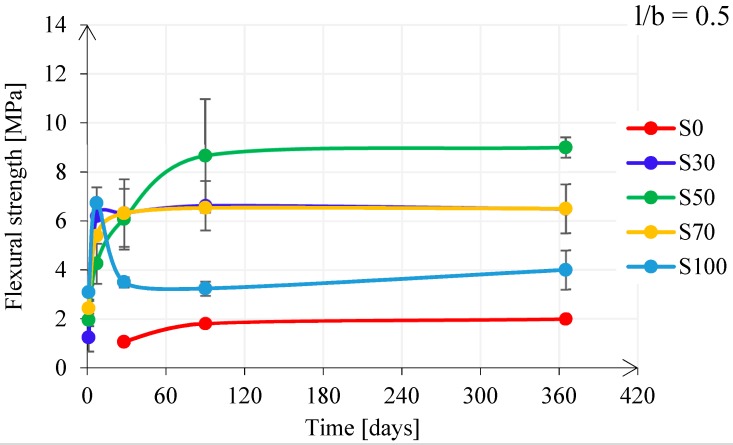
Flexural strength development for pastes with l/b = 0.5.

Figure 14 shows the influence of the FA/GBFS ratio on the flexural strength development for pastes with l/b = 0.4. The flexural strength had a similar behaviour for pastes S30, S50, and S100, while paste S0 and paste S70 showed a different behaviour. Similar to the compressive strength development, paste S0 had a slow development of the flexural strength. For pastes with GBFS, the scatter was significant and there was only an increasing tendency for paste S70. The flexural strength development for pastes with l/b = 0.5 had different behaviour than for pastes with l/b = 0.4 (see Figure 15). It increased for pastes S30, S50, S70, while it decreased only for paste S100. The scatter among different pastes with l/b = 0.5 was less noticeable in comparison with that in pastes with l/b = 0.4. 

**Figure 16:**
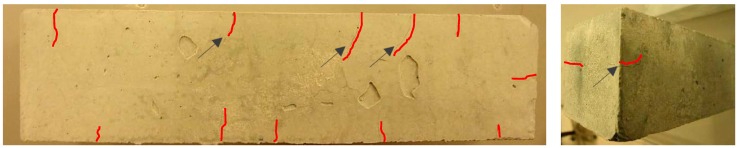
Visual observations of sample S100 surfaces. Samples were cured for 14 days (fog room, at 20 °C and 99% RH) and subsequently removed to laboratory conditions, at 20 °C and 55% RH. Arrows point to microcracks patterns, which are coloured in red. (The microcracks were most present on the top surface, while on the other sides of samples their appearance was less).

Large deviations of flexural strength evolution appeared in all alkali activated GBFS pastes, specifically for the lower l/b ratio (see Figure 14). The large scatter is mainly due to the development of microcracks, which were observed visually and microscopically. Microcracks were present at both surfaces of the sample (Figure 16), but also inside the sample, as also observed by Collins and Sanjayan [74], Ye et al. [75], Thomas et al. [71], and Hubler et al. [76]. Nevertheless, microcracks did not influence the compressive strength. The larger the amount of reaction products, with the elapse of time, resulted in higher compressive strength (see Figure 11 and Figure 12). However, when the pastes were tested during bending, microcracks played a significant role. In particular, the flexural fracture of pastes could be affected due to small defects, such as surface drying shrinkage-induced and autogenous shrinkage-induced microcracking, compared to the mortar or even concrete, where these defects were normally reduced due to the presence of aggregates and sample size. The autogenous shrinkage amplitudes will be investigated in next section. The microcracks in GBFS-rich pastes, if connected, could form preferential continuous pathways for mass transport and faster propagation of the water or other compounds (e.g., O_2_ and CO_2_). For this reason, microcracks were expected to influence not only the flexural strength but also the permeability of the material.

Beside shrinkage-induced microcracking, the “defect density of unreacted material” could also potentially be an explanation for the observed flexural strength behaviour. The concept has been proposed by Duxson et al. [77] for metakaolin-based geopolymers with various Si/Al ratios. It is related to the gel transformation and densification during geopolymerization. It is believed that the defect density increases with an increase in the amount of unreacted material (which is a potential reason for the larger scatter of the results with l/b = 0.4 compared to the results with l/b = 0.5, since a larger amount of unreacted material was assumed to be present in pastes with a lower l/b ratio). Consequently, with an increased defect density, the number of potential pathways to failure increased accordingly. In addition, it was found that more labile species present in the pore solution and gel nanostructure of AAMs with lower SiO_2_/Na_2_O ratio allowed for a greater degree of structural reorganization and densification of the gel units prior to hardening [77]. The structural ordering of these gel units, their interconnectivity, and their morphological, physical, and chemical changes over time play a critical role in understanding the mechanical strength evolution. Further research is needed to investigate and quantify this behaviour in alkali-activated FA/GBFS materials.

### 3.5. Autogenous Shrinkage

**Figure 17:**
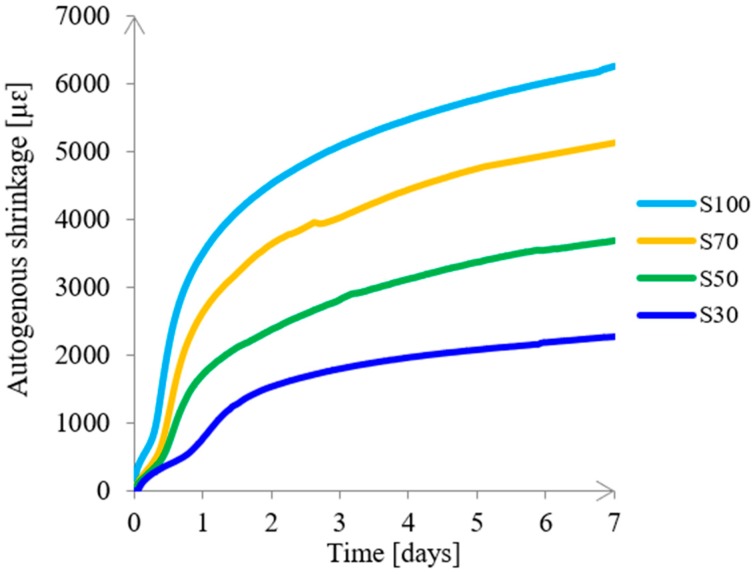
Autogenous shrinkage of alkali activated pastes (l/b = 0.5).

According to the study of Ma and Ye [78], the autogenous shrinkage of pure FA-based alkali-activated paste does not result in microcracking. Therefore, the autogenous shrinkage of paste S0 was not measured in this study. Since l/b = 0.5 improved the workability of the paste without harming the strength, the autogenous shrinkage of pastes with an l/b of 0.5 during the first 7 days was further explored. As shown in Figure 17, mixture S100 had the highest autogenous shrinkage, reaching more than 6000 microstrains at 7 days. On average, this value was 10 times higher than of cement paste [29]. Namely, the formation of a large amount of reaction products in paste S100, as shown in Figure 9b, caused large self-desiccation and high capillary pressure. These two factors were the main driving mechanisms for such a high autogenous shrinkage [79]. The autogenous shrinkage decreased with a decrease of GBFS. The shrinkage amplitude of 3700 microstrains was reached after 1 day for paste S100, and at 7 days for paste S50. The activation of FA led to a lower autogenous shrinkage due to the use of ambient temperature for curing of the specimens. This curing condition caused a low degree of FA reaction [33], supported by low strength, as shown in Figure 12. It was assumed that high autogenous shrinkage induces stresses and finally microcracks. This affected the flexural strength results, as discussed in the previous section. The microcracks were expected to influence not only the flexural strength but also the transport properties of the material. Since high autogenous shrinkage may induce microcracking, the mixtures with low GBFS content are recommended for further applications.

## 4. Conclusions

The aim of this paper was to obtain mixtures with adequate workability, strength, and autogenous shrinkage. The fresh properties and mechanical performance of the pastes have been studied for different l/b ratios, different FA/GBFS ratios, and a constant alkaline activator composition. Based on the experimental results and observations, the following conclusions can be drawn:

- The FA/GBFS ratio of the mixture significantly affected the workability, setting time, and mechanical properties of alkali-activated pastes. Results showed that all pastes, except pure FA paste (S0), exhibited high reaction rates and high mechanical strength due to large amorphous content in both GBFS and FA.
- By studying the influence of different l/b, it was found that mixtures with l/b = 0.4 had a faster initial setting time and stiffer consistency than mixtures with l/b = 0.5. The l/b ratio did not significantly affect the compressive strength development. However, l/b = 0.5 improved the workability and provided a longer initial setting time for the pastes, which is one of the main criteria for casting concrete.
- Regarding the rate of heat evolution of the mixtures, the pure GBFS (S100) had the highest rate of heat release, subsequently developing higher compressive strength and autogenous shrinkage than other mixtures. Moreover, GBFS-rich mixtures had shorter setting times due to the high reactivity of GBFS, irrespective of the l/b ratio.
- The flexural strength varied to a great extent with the type of paste mixture. With regard to the scatter, it was assumed that shrinkage-induced microcracking and “defect density of unreacted material” were the main reasons for such flexural strength behaviour. These observations were essential for the understanding of the macroscopic structural behaviour of AAMs, which depends on the mechanical behaviour of its constituents at smaller scales, e.g., paste and paste interfaces with sand particles. Further research is needed to confirm these mechanisms in alkali activated FA and GBFS materials.
- For the pastes with an l/b of 0.5, it was found that the GBFS-rich mixtures showed a higher autogenous shrinkage due to the more severe self-desiccation induced by the reactions of GBFS. The mixtures with an l/b of 0.5 and relatively low GBFS content (such as S30 and S50) exhibited better workability, acceptable strength, and lower autogenous shrinkage, thus are recommended for future engineering applications.

## Figures and Tables

**Table 1:** Chemical composition of GBFS and FA measured with XRF [%].

Raw Material	SiO_2_	Al_2_O_3_	CaO	MgO	Fe_2_O_3_	S	Na_2_O	K_2_O	TiO_2_	P_2_O_5_	LOI
GBFS	35.5	13.5	39.8	8.0	0.64	1.0	0.4	0.53	1.0	0.00	−1.3
FA	56.8	23.8	4.8	1.5	7.20	0.3	0.8	1.60	1.2	0.51	1.2

**Table 2:** Mixture design with respect to 100 g of binder.

Mixture	FA	GBFS	CEM I	m(SiO_2_)/ m(Na_2_O)	m(Na_2_O)/ m(binder)	l/b (i)	l/b (ii)
S0	100	0	-	1.45	4.8	0.4	0.5
S30	70	30					
S50	50	50					
S70	30	70					
S100	0	100					
CEM I			100	-	-		

**Table 3:** Properties of gel phases in alkali-activated GBFS paste and OPC-based paste.

Property	Alkali-Activated GBFS, w/GBFS = 0.38	OPC Paste [70], w/OPC = 0.35
Ca/Si	0.84	1.4–1.6
Gel morphology	Foil-like	Fiber or honeycomb-like
Gel alkali binding capacity [75]	Moderate	Low
SANS specific surface area [71]	High	Low
Atomic packing density [71]	High	Low
Amorphous phase content at 28 days	99 wt%	76.2 wt%
Compressive strength at 28 days	112.7 MPa	77.6 MPa
Compressive strength at 60 days	113.0 MPa (maximum)	84.5 MPa (maximum)

## References

[1] Huntzinger D.N., Eatmon T.D. (2009). A life-cycle assessment of Portland cement manufacturing: Comparing the traditional process with alternative technologies. J. Clean. Prod..

[2] https://www.iea.org/publications/freepublications/publication/Cement.pdf.

[3] McLellan B.C., Williams R.P., Lay J., Van Riessen A., Corder G.D. (2011). Costs and carbon emissions for geopolymer pastes in comparison to ordinary portland cement. J. Clean. Prod..

[4] Habert G., Ouellet-Plamondon C. (2016). Recent update on the environmental impact of geopolymers. RILEM Tech. Lett..

[5] Schneider M., Romer M., Tschudin M., Bolio H. (2011). Sustainable cement production—Present and future. Cem. Concr. Res..

[6] Worrell E., Price L., Martin N., Hendriks C., Meida L.O. (2001). Carbon dioxide emissions from the global cement industry. Annu. Rev. Energy Environ..

[7] Miller S.A., Horvath A., Monteiro P.J. (2016). Readily implementable techniques can cut annual CO_2_ emissions from the production of concrete by over 20%. Environ. Res. Lett..

[8] Scharff H. (2014). Landfill reduction experience in The Netherlands. Waste Manag..

[9] Lothenbach B., Scrivener K., Hooton R. (2011). Supplementary cementitious materials. Cem. Concr. Res..

[10] Siddique R., Khan M.I. (2011). Supplementary Cementing Materials.

[11] Van Deventer J.S., Provis J.L., Duxson P., Brice D.G. (2010). Chemical research and climate change as drivers in the commercial adoption of alkali activated materials. Waste Biomass Valoriz..

[12] Davidovits J. (1991). Geopolymers. J. Therm. Anal..

[13] Palomo A., Grutzeck M.W., Blanco M.T. (1999). Alkali-activated fly ashes: A cement for the future. Cem. Concr. Res..

[14] Krizan D., Zivanovic B. (2002). Effects of dosage and modulus of water glass on early hydration of alkali–slag cements. Cem. Concr. Res..

[15] Van Jaarsveld J.G.S., van Deventer J.S.J., Lukey G.C. (2003). The characterisation of source materials in fly ash-based geopolymers. Mater. Lett..

[16] Criado M., Palomo A., Fernández-Jiménez A. (2005). Alkali activation of fly ashes. Part 1: Effect of curing conditions on the carbonation of the reaction products. Fuel.

[17] Škvára F., Jílek T., Kopecký L. (2005). Geopolymer materials based on fly ash. Ceram. Silik..

[18] Chancey R.T., Stutzman P., Juenger M.C., Fowler D.W. (2010). Comprehensive phase characterization of crystalline and amorphous phases of a Class F fly ash. Cem. Concr. Res..

[19] Durdziński P.T., Dunant C.F., Haha M.B., Scrivener K.L. (2015). A new quantification method based on SEM-EDS to assess fly ash composition and study the reaction of its individual components in hydrating cement paste. Cem. Concr. Res..

[20] Provis J.L., Hajimohammadi A., White C.E., Bernal S.A., Myers R.J., Winarski R.P., Rose V., Proffen T.E., Llobet A., van Deventer J.S. (2013). Nanostructural characterization of geopolymers by advanced beamline techniques. Cem. Concr. Compos..

[21] Glukhovsky V. (1959). Soil Silicates.

[22] Glukhovskij V., Zaitsev Y., Pakhomov V. (1983). Slag-alkaline cements and concretes-structure, properties, technological and economical aspects of the use. Silic. Indus..

[23] Richardson I.G., Brough A.R., Groves G.W., Dobson C.M. (1994). The characterization of hardened alkali-activated blast-furnace slag pastes and the nature of the calcium silicate hydrate (C-S-H) phase. Cem. Concr. Res..

[24] Brough A.R., Holloway M., Sykes J., Atkinson A. (2000). Sodium silicate-based alkali-activated slag mortars: Part II. The retarding effect of additions of sodium chloride or malic acid. Cem. Concr. Res..

[25] Xu H., Van Deventer J.S.J. (2000). The geopolymerisation of alumino-silicate minerals. Int. J. Miner. Process..

[26] Phair J.W., Van Deventer J.S.J. (2001). Effect of silicate activator pH on the leaching and material characteristics of waste-based inorganic polymers. Miner. Eng..

[27] Dembovska L., Bajare D., Ducman V., Korat L., Bumanis G. (2017). The use of different by-products in the production of lightweight alkali activated building materials. Constr. Build. Mater..

[28] Puertas F., Fernández-Jiménez A., Blanco-Varela M. (2004). Pore solution in alkali-activated slag cement pastes. Relation to the composition and structure of calcium silicate hydrate. Cem. Concr. Res..

[29] García-Lodeiro I., Palomo A., Fernández-Jiménez A., Macphee D.E. (2011). Compatibility studies between N-A-S-H and C-A-S-H gels. Study in the ternary diagram Na_2_O–CaO–Al_2_O_3_–SiO_2_–H_2_O. Cem. Concr. Res..

[30] Němeček J., Šmilauer V., Kopecký L. (2011). Nanoindentation characteristics of alkali-activated aluminosilicate materials. Cem. Concr. Compos..

[31] Provis J.L., Rose V., Winarski R.P., van Deventer J.S. (2011). Hard X-ray nanotomography of amorphous aluminosilicate cements. Scr. Mater..

[32] Rovnaník P. (2010). Effect of curing temperature on the development of hard structure of metakaolin-based geopolymer. Constr. Build. Mater..

[33] Ma Y. (2013). Microstructure and Engineering Properties of Alkali Activated Fly Ash; as an Environment Friendly Alternative to Portland Cement.

[34] Provis J.L., Duxson P., van Deventer J.S.J. (2010). The role of particle technology in developing sustainable construction materials. Adv. Powder Technol..

[35] Nedeljković M., Arbi K., Zuo Y., Ye G. Physical properties and pore solution analysis of alkali activated fly ash-slag pastes. Proceedings of the International RILEM Conference on Materials, Systems and Structures in Civil Engineering, Conference segment on Concrete with Supplementary Cementitious materials.

[36] Van Deventer J.S.J., Provis J.L., Duxson P. (2012). Technical and commercial progress in the adoption of geopolymer cement. Miner. Eng..

[37] Arbi K., Nedeljković M., Zuo Y., Ye G. (2016). A review on the durability of alkali-activated fly ash/slag systems: Advances, issues, and perspectives. Ind. Eng. Chem. Res..

[38] Shi C., Roy D., Krivenko P. (2003). Alkali-Activated Cements and Concretes.

[39] Wang S.D., Scrivener K.L., Pratt P.L. (1994). Factors affecting the strength of alkali-activated slag. Cem. Concr. Res..

[40] Duxson P., Fernández-Jiménez A., Provis J.L., Lukey G.C., Palomo A., van Deventer J.S. (2007). Geopolymer technology: The current state of the art. J. Mater. Sci..

[41] Sindhunata, Van Deventer J.S.J., Lukey G.C., Xu H. (2006). Effect of Curing Temperature and Silicate Concentration on Fly-Ash-Based Geopolymerization. Ind. Eng. Chem. Res..

[42] Pacheco-Torgal F., Labrincha J., Leonelli C., Palomo A., Chindaprasit P. (2014). Handbook of Alkali-Activated Cements, Mortars and Concretes.

[43] Chang J.J. (2003). A study on the setting characteristics of sodium silicate-activated slag pastes. Cem. Concr. Res..

[44] Nath P., Sarker P.K. (2014). Effect of GGBFS on setting, workability and early strength properties of fly ash geopolymer concrete cured in ambient condition. Constr. Build. Mater..

[45] Palacios M., Banfill P.F., Puertas F. (2008). Rheology and setting of alkali-activated slag pastes and mortars: Effect of organic admixture. ACI Mater. J..

[46] Haha M.B., Le Saout G., Winnefeld F., Lothenbach B. (2011). Influence of activator type on hydration kinetics, hydrate assemblage and microstructural development of alkali activated blast-furnace slags. Cem. Concr. Res..

[47] Wardhono A., Gunasekara C., Law D.W., Setunge S. (2017). Comparison of long term performance between alkali activated slag and fly ash geopolymer concretes. Constr. Build. Mater..

[48] Jensen O.M., Hansen P.F. (2001). Autogenous deformation and RH-change in perspective. Cem. Concr. Res..

[49] Li Z., Liu J., Ye G. Drying shrinkage of alkali-activated slag and fly ash concrete. A comparative study with ordinary Portland cement concrete. Proceedings of the Workshop on Concrete Modelling and Materials Behaviour in honor of Professor Klaas van Breugel.

[50] Wan H., Shui Z., Lin Z. (2004). Analysis of geometric characteristics of GGBS particles and their influences on cement properties. Cem. Concr. Res..

[51] Wang P., Trettin R., Rudert V. (2005). Effect of fineness and particle size distribution of granulated blast-furnace slag on the hydraulic reactivity in cement systems. Adv. Cem. Res..

[52] Marinković S.B., Ignjatović I.S., Dragaš J.S., Tošić N.D., Nedeljković M.R. Experimental study of alkali activated fly ash concrete with fly ash from one serbian power plant. Proceedings of the International Conference on Sustainable Structural Concrete.

[53] Panias D., Giannopoulou I.P., Perraki T. (2007). Effect of synthesis parameters on the mechanical properties of fly ash-based geopolymers. Colloids Surf. A.

[54] Douglas E., Bilodeau A., Brandstetr J., Malhotra V.M. (1991). Alkali activated ground granulated blast-furnace slag concrete: Preliminary investigation. Cem. Concr. Res..

[55] Burciaga-Díaz O., Escalante-García J.I., Arellano-Aguilar R., Gorokhovsky A. (2010). Statistical analysis of strength development as a function of various parameters on activated metakaolin/slag cements. J. Am. Ceram. Soc..

[56] Tan Z., Bernal S.A., Provis J.L. (2017). Reproducible mini-slump test procedure for measuring the yield stress of cementitious pastes. Mater. Struct..

[57] Jiang S., Mutin J., Nonat A. (1995). Studies on mechanism and physico-chemical parameters at the origin of the cement setting. I. The fundamental processes involved during the cement setting. Cem. Concr. Res..

[58] Bentz D.P. (2007). Cement hydration: Building bridges and dams at the microstructure level. Mater. Struct..

[59] (1995). Methods of Testing Cement—Part 3: Determination of Setting Times and Soundness.

[60] Zhang M.H., Sisomphon K., Ng T.S., Sun D.J. (2010). Effect of superplasticizers on workability retention and initial setting time of cement pastes. Constr. Build. Mater..

[61] (2014). Standard Practice for Measuring Hydration Kinetics of Hydraulic Cementitious Mixtures Using Isothermal Calorimetry.

[62] (2005). Methods of Testing Cement—Part 1: Determination of Strength.

[63] Jiao D., Shi C., Yuan Q., An X., Liu Y., Li H. (2017). Effect of constituents on rheological properties of fresh concrete-A review. Cem. Concr. Compos..

[64] Pólya G., Szego J.N., Szegő G. (1951). Isoperimetric Inequalities in Mathematical Physics.

[65] Taylor H.F. (1997). Cement Chemistry.

[66] Fernandez-Jimenez A.M., Palomo A., Lopez-Hombrados C. (2006). Engineering properties of alkali-activated fly ash concrete. ACI Mater. J..

[67] Trtnik G., Turk G., Kavčič F., Bosiljkov V.B. (2008). Possibilities of using the ultrasonic wave transmission method to estimate initial setting time of cement paste. Cem. Concr. Res..

[68] Li S., Sha F., Liu R., Zhang Q., Li Z. (2017). Investigation on fundamental properties of microfine cement and cement-slag grouts. Constr. Build. Mater..

[69] Brough A.R., Atkinson A. (2002). Sodium silicate-based, alkali-activated slag mortars: Part I. Strength, hydration and microstructure. Cem. Concr. Res..

[70] Chindaprasirt P., Jaturapitakkul C., Sinsiri T. (2005). Effect of fly ash fineness on compressive strength and pore size of blended cement paste. Cem. Concr. Compos..

[71] Thomas J.J., Allen A.J., Jennings H.M. (2012). Density and water content of nanoscale solid C–S–H formed in alkali-activated slag (AAS) paste and implications for chemical shrinkage. Cem. Concr. Res..

[72] Knudsen F. (1959). Dependence of mechanical strength of brittle polycrystalline specimens on porosity and grain size. J. Am. Ceram. Soc..

[73] Nedeljković M., Ghiassi B., Van der Laan S., Li Z., Ye G. Effect of curing conditions on the pore solution and carbonation resistance of alkali-activated fly ash and slag pastes. Cem. Concr. Res..

[74] Collins F., Sanjayan J. (2001). Microcracking and strength development of alkali activated slag concrete. Cem. Concr. Compos..

[75] Ye H., Radlińska A. (2017). Effect of Alkalis on Cementitious Materials: Understanding the Relationship between Composition, Structure, and Volume Change Mechanism. J. Adv. Concr. Technol..

[76] Hubler M.H., Thomas J.J., Jennings H.M. (2011). Influence of nucleation seeding on the hydration kinetics and compressive strength of alkali activated slag paste. Cem. Concr. Res..

[77] Duxson P., Provis J.L., Lukey G.C., Mallicoat S.W., Kriven W.M., Van Deventer J.S. (2005). Understanding the relationship between geopolymer composition, microstructure and mechanical properties. Colloids Surf. A.

[78] Ma Y., Ye G. (2015). The shrinkage of alkali activated fly ash. Cem. Concr. Res..

[79] Li Z., Nedeljković M., Zuo Y., Ye G. Autogenous shrinkage of alkali-activated slag-fly ash pastes. Proceedings of the 5th International Slag Valorisation Symposium.

